# Highly Sensitive, Breathable, and Flexible Pressure Sensor Based on Electrospun Membrane with Assistance of AgNW/TPU as Composite Dielectric Layer

**DOI:** 10.3390/s20092459

**Published:** 2020-04-26

**Authors:** Jie Wang, Yaoyuan Lou, Bin Wang, Qing Sun, Mingwei Zhou, Xiuyan Li

**Affiliations:** 1 School of Materials Science & Engineering, Beijing Institute of Fashion Technology, Beijing 100029, China; 15313719909@163.com (J.W.); clylxyan@bift.edu.cn (X.L.); 2 Beijing Key Laboratory of Clothing Materials R & D and Assessment, Beijing Engineering Research Center of Textile Nanofiber, Beijing Institute of Fashion Technology, Beijing 100029, China; 3 College of Materials Science and Engineering, Zhejiang University of Technology, Hangzhou 310014, Zhejiang, China

**Keywords:** capacitive pressure sensor, electrospinning nanofiber membrane, high sensitivity, wearing comfortability, composite dielectric layer

## Abstract

Pressure sensors have been widely used in electronic wearable devices and medical devices to detect tiny physical movements and mechanical deformation. However, it remains a challenge to fabricate desirable, comfortable wearing, and highly sensitive as well as fast responsive sensors to capture human body physiological signs. Here, a new capacitive flexible pressure sensor that is likely to solve this problem was constructed using thermoplastic polyurethane elastomer rubber (TPU) electrospinning nanofiber membranes as a stretchable substrate with the incorporation of silver nanowires (AgNWs) to build a composite dielectric layer. In addition, carbon nanotubes (CNTs) were painted on the TPU membranes as flexible electrodes by screen printing to maintain the flexibility and breathability of the sensors. The flexible pressure sensor could detect tiny body signs; fairly small physical presses and mechanical deformation based on the variation in capacitance due to the synergistic effects of microstructure and easily altered composite permittivity of AgNW/TPU composite dielectric layers. The resultant sensors exhibited high sensitivity (7.24 kPa^−1^ within the range of 9.0 × 10^−3^ ~ 0.98 kPa), low detection limit (9.24 Pa), and remarkable breathability as well as fast responsiveness (<55 ms). Moreover, both continuously pressing/releasing cycle over 1000 s and bending over 1000 times did not impair the sensitivity, stability, and durability of this flexible pressure sensor. This proposed strategy combining the elastomer nanofiber membrane and AgNW dopant demonstrates a cost-effective and scalable fabrication of capacitive pressure sensors as a promising application in electronic skins and wearable devices.

## 1. Introduction 

Pressure sensors that transform external stimulation to electronic signals has received extensive attention in the past few years, with typical sensing methods such as transistor pressure sensors [1], piezoelectric pressure sensors [2,3,4,5], capacitive pressure sensors [6,7], piezoresistive pressure sensors [8,9,10,11] and triboelectric pressure sensors [12,13]. Among them, capacitive pressure sensors have been widely applied due to their easy design and preparation, high sensitivity, and cost-effective features. Currently, with the development of technology and the progress of society, more and more flexible and highly sensitive pressure sensors are being developed for various applications in health monitoring [14,15], human-machine interfaces [16,17], smart robots [18,19], wearable devices [20,21], etc. In designing capacitive pressure sensors that imitate human skin, high sensitivity, good reproducibility, and excellent wearing comfort have always been desirable characteristics that researchers highly pursue [22]. 

Although capacitive pressure sensors with high sensitivity and good flexibility have been investigated, the current performances of on-skin sensors show that it is difficult to satisfy the needs of accurate and continuous physiological detection over a long period of time [23]. To address this challenge, constructing various microstructures onto a pressure sensing layer was first thought of by researchers such as pyramids, lotus leaf, and gradient wrinkles [24,25,26]. For example, Shuai et al. fabricated a highly sensitive flexible pressure sensor based on elastomeric electrodes and a microarray architecture [27]. A polydimethylsiloxane (PDMS) substrate, coated with silver nanowires (AgNWs), was used as the top electrode, while polyvinylidene fluoride (PVDF) was the dielectric layer. The flexible pressure sensor integrated the top electrode, dielectric layer, and microarray electrode in a sandwich structure and demonstrated that such sensors possess superiorities in high sensitivity (2.94 kPa^−1^), low detection limit (<3 Pa), short response time (<50 ms), excellent flexibility, and long-term cycle stability (1000 cycles). Apart from the microstructure construction, the sensing performance of a capacitive pressure sensor can be greatly improved by increasing the permittivity of the dielectric layer [28]. There are a variety of nanomaterials that have been involved such as reduced graphene oxide (rGO) [29], carbon nanotubes (CNTs) [30], and AgNWs [31]. For example, Jia et al. [32] managed to fabricate a capacitive pressure sensor by using rGO film as the dielectric layer to increase the permittivity, which not only achieved high sensitivity (178 kPa^−1^), but also acquired a low detection limit (42 Pa). Comparatively speaking, AgNWs display synergetic advantages in being able to change the permittivity of dielectric layer and construct a microstructure in the pressure sensing layer due to the high length–diameter ratio. Shi et al. [33] successfully fabricated a flexible transparent capacitive pressure sensor based on patterned microstructure AgNW/PDMS composite dielectrics. Compared with the pure PDMS dielectric layer with planar structures, the patterned microstructure sensor exhibited a higher sensitivity (0.831 kPa^−1^, <0.5 kPa), a lower detection limit (1.4 Pa), and a shorter response time (<30 ms) and relaxation time (<60 ms). 

Although these developments have greatly improved the sensing performance of capacitive sensors, the harsh preparation method and complicated multistep hinder the scale production of highly-sensitive capacitive pressure sensors. Moreover, the dependence an on airtight substrate sacrifices the wearing comfort, thus the skin cannot breathe when it attached to the human body, resulting in skin swelling and/or inflammation after wearing for a long times [34]. Hence, a breathable, lightweight, stretchable substrate with tunable microstructure and permittivity should promote the sensing performance and long-term comfort of on-skin pressure sensors. Electrospinning nanofiber membranes with inherently high gas-permeability, excellent flexibility, and abundant microstructures could be more desirable for on-skin sensors in monitoring vital signals and motions under normal, everyday conditions. In our previous research [35], we reported a breathable capacitive pressure sensor with good sensitivity of 4.2 kPa^−1^ based on a sandwich structure, which was composed of two AgNW/poly(vinylidene fluoride) (PVDF) nanofiber membrane electrodes and a thermoplastic polyurethane elastomer (TPU) nanofiber membrane dielectric layer. However, the flexibility and sensitivity are still unsatisfying. 

Here, a highly-sensitive and durable capacitive pressure sensor, which was completely assembled by elastomer electrospinning nanofiber membranes with excellent breathability and flexibility, was successfully fabricated by electrospinning technology and the screen printing method. A mesh structure made of TPU electrospinning nanofiber membranes (ENMs) avoids causing discomfort, even it is fixed onto the skin for a long time. In addition, it also introduces AgNWs as permittivity modifiers into the TPU nanofiber membranes to construct a composite dielectric layer. The synergistic effect of the nanofibrous microstructure of the TPU membrane and incorporation of AgNWs into the dielectric layer not only provides a high sensitivity of 7.24 kPa^−1^ in a low-pressure range (9.0 × 10^−3^ ~ 0.98 kPa), but also improves the response time and durability. Therefore, the facile preparation technique and the positive characteristics such as high sensitivity, excellent breathability, and durability mean that this nanofiber membrane based flexible capacitive pressure sensor shows great potential for applications in wearable sensing devices. 

## 2. Experimental 

### 2.1. Material

The thermoplastic polyurethane (TPU) elastomer was obtained from BASFCo., Ltd. (Ludwigshafen, Germany), ethylene glycol (EG), acetone, N, N-dimethylformamide (DMF), tetrahydrofuran (THF), silver nitrate (AgNO_3_), sodium chloride (NaCl), and polyvinyl pyrrolidone (PVP, K-30) were purchased from Beijing Chemical Reagents Co., Ltd. (Beijing, China). Deionized water was prepared in our laboratory. Carbon nanotube aqueous dispersion, as an additive of the electrode material, was produced in XFNANO Co., Ltd. (Nanjing, China). 

### 2.2. Preparation and Assembly of Flexible Capacitive Pressure Sensor Based on Nanofiber Membrane

#### 2.2.1. Preparation of Silver Nanowires (AgNWs)

**Figure 1:**
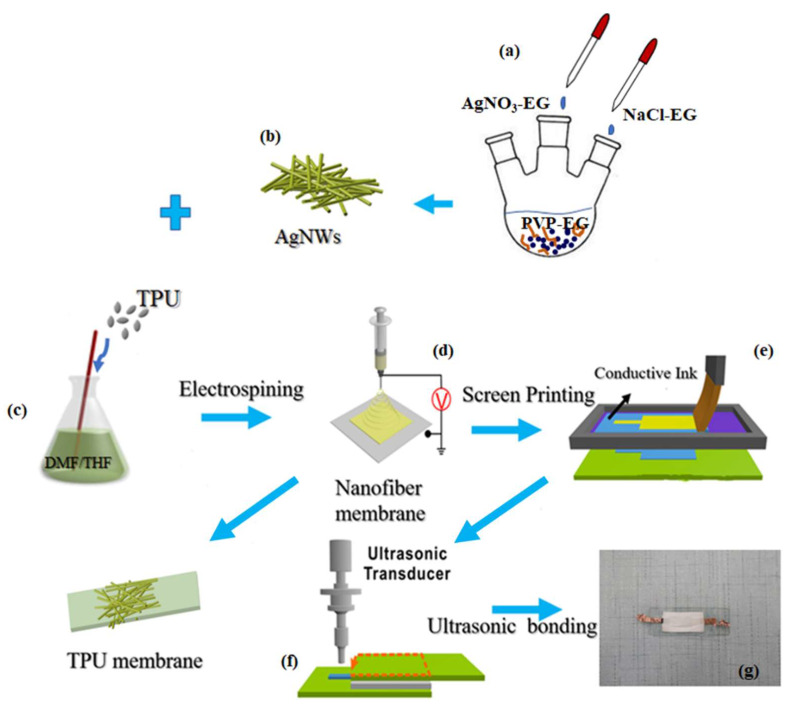
Fabrication schematics for the nanofiber membrane based flexible capacitive pressure sensor: (**a**,**b**) preparation of silver nanowires (AgNWs), (**c**) preparation of spinning solution, (**d**) electrospinning process, (**e**–**g**) assembly of sensors: electrode printing and ultrasonic welding.

The AgNWs were synthesized by the polyol process. First, 3.03 g AgNO_3_ was dissolved in 9.8 g EG by stirring for 1 h at room temperature to form a AgNO_3_–EG solution. Then, 0.116 g NaCl was added to 10 mL EG and stirred well until the formation of the NaCl–EG solution. Next, 2.949 g PVP was dissolved in 253.7 g EG by stirring at 160 °C for 50 min in a flask. A solution of 480 μL NaCl-EG was added to the flask and 10 mL AgNO_3_–EG solution was added at 1.2 mL/min. When the reaction solution started to glisten, the synthetic reaction was continued for another hour at 160 °C. Finally, the obtained AgNWs were washed with acetone to remove the residual PVP and collected for later use. The preparation process of AgNWs is shown in Figure 1a,b. 

#### 2.2.2. Fabrication of Thermoplastic Polyurethane (TPU) Electrospinning Nanofiber Membranes (ENMs) and AgNW/TPU ENMs by Electrospinning

First, 5 g of TPU was dissolved in 20 mL mixture solutions with THF (10 mL) and DMF (10 mL) under magnetic stirring until a uniform solution was obtained. Then, using the electrospinning technology to prepare TPU ENM at a high electric potential of 20 kV, the feed rate of the TPU solution was fixed at 1.0 mL/h by using an injection pump. The collecting distance between needle tips and the drum that served as a collector was 10 cm. After different electrospinning times, a series of TPU ENMs with varying thicknesses were obtained and named as TPU-*x* ENMs (*x* = 1 h, 2 h, 3 h, 4 h, and 5 h). For the AgNW/TPU composite nanofiber membranes, we added AgNWs of different quantities to the TPU electrospinning precursor that was then spun under the same conditions as the TPU ENMs. In the same way, we named TPUENMs incorporated with AgNWs as AgNW/TPU-*x* ENMs (*x* = 1.0 mL, 2.0 mL, 3.0 mL, 4.0 mL, and 5.0 mL). The fabrication of the AgNW/TPU composite nanofiber membrane is shown in Figure 1.

#### 2.2.3. Design and Assembly of Flexible Capacitive Pressure Sensor

According to our previous work [36], we used a screen printing technique and ultrasonic bonding method to construct and package TPU nanofiber membrane based flexible capacitive pressure sensors, as illustrated in Figure 1. First, we used the prepared TPU ENMs as the supporting layer and printed conductive CNT ink onto them as the electrode layers. The prepared AgNW/TPU ENMs with different amounts of AgNWs were in the middle of the sensor as a dielectric layer. Finally, the middle AgNW/TPU ENM dielectric layer and bottom and top TPU electrode layers were face-to-face assembled into sensors through ultrasonic bonding (OL-1526, Dongguan City South Nekon Machinery Co., Ltd. (Dongguan, China). 

### 2.3. Characterization

The surface morphology and microstructure of the nanofiber membranes were observed with a scanning electron microscope (JSM 7500, JEOL, (Tokyo, Japan). Energy-dispersive X-ray spectroscopy (EDS) was employed to confirm the incorporation and distribution of AgNWs in the nanofiber membrane. X-ray diffractometer (XRD) studies were conducted on a Rigaku D/Max-Ultima III X-ray diffractometer equipped with a Cu-Kα source (40 kV, 200 mA). A thickness gauge (CHY-CB, Jinan Lab think electromechanical technology, (Jinan, China) was used to measure the thickness of the nanofiber membrane and a dynamic/static water contact goniometer (SL200B, Kono industry, Houston, USA) was used to test the hydrophobic property of the nanofiber membranes. An electronic universal testing machine (EZ-LX, Shimadzu, Osaka, Japan) was used to test the mechanical property of the nanofiber membranes. An automatic air permeability tester (YG4G12, Laizhou Electron Instrument, Yantai, China) was used to test the breathability of the nanofiber membranes. The capacitance variation of the sensor was carried out by a LCR meter, which is a special measuring tool for measuring parameters of electrical components. L: inductance, C: Capacitance, R: Resistance. (E4980AL, Agilent, Palo Alto, USA). 

## 3. Results and Discussion

### 3.1. Morphology and Properties of TPU ENMs and AgNW/TPU ENMs

**Figure 2:**
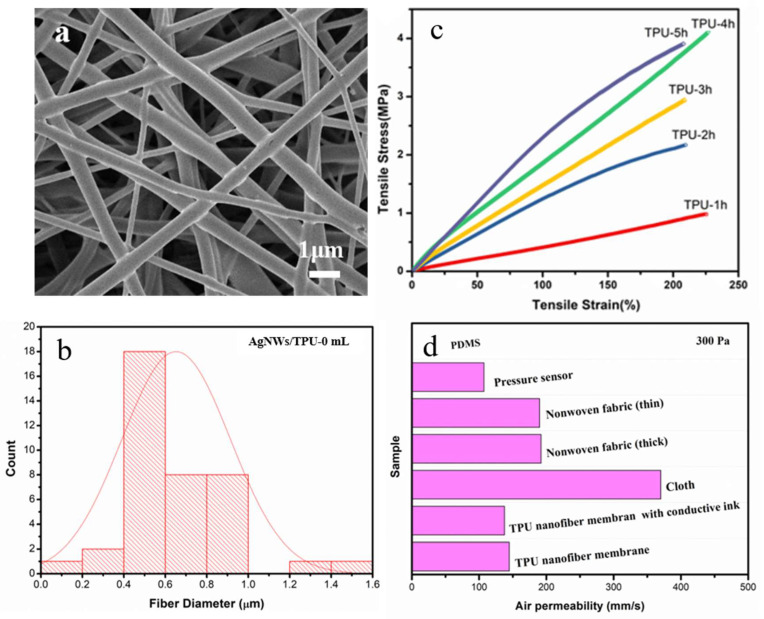
The morphology and partial performance test of the TPU ENMs: (**a**,**b**) SEM and diameter distribution of the TPU ENMs, (**c**) mechanical properties of TPU ENMs with different spinning time, (**d**) comparison of breathability.

TPU ENMs and AgNW/TPU ENMs were studied in terms of their morphology, composition, breathability, and mechanical properties to understand and evaluate the synergistic effects of nanofibrous microstructures and AgNW doping on the sensing performance of the TPU nanofiber based flexible capacitive pressure sensor. The morphology of the TPU ENMs with different spinning times are shown in Figure S1 (Supplementary Materials). As shown in Figure 2a,b, as the fiber diameter was uniform and the average diameter was 652 ± 5.0 nm. Additionally, there were no spindles or breakages in the TPU-3 h ENMs, which is beneficial to the flexibility and reproducibility of sensor. 

Since mechanical properties such as tensile strength, tensile strain, and elongation at break are essential factors for flexible pressure sensors, we determined the mechanical properties of the TPU nanofiber membranes according to the test process shown in Figure S2a–c. The mechanical properties of the samples were primarily based on the nanofiber’s diameter distribution and thickness. The tensile strain of the TPU-3 h ENMs was about 200% and the tensile stress was 2.8 MPa. It can clearly be seen from Figure 2c that the slope of the stress–strain curve, namely the stiffness of the TPU ENMs, increased with the extension of spinning time due to the increase in the thickness of the nanofiber membrane. Moreover, the stiffness and thickness of the nanofiber membrane will affect the final sensing performance (particularly sensitivity). In terms of balancing the tensile strength and sensing performance, we believe that a more suitable spinning time is three hours. 

In addition, under the pressure drop of 300 Pa, we tested and compared the air permeability of TPU ENMs with other sensor substrates and common fabrics, and the results are shown in Figure 2d. The air permeability of the TPU-3h ENMs was 138 mm/s, which was better than that of the PDMS membrane (0 mm/s) commonly used for flexible pressure sensors [37,38]. Furthermore, the sandwich structured flexible pressure sensor based on the TPU ENMs had a permeability of 120 mm/s, which was similar to ordinary nonwoven fabrics (about 190 mm/s). This important and interesting result ensures that the sweat evaporation of skin will not be hindered by attaching this sensor, thus improving the wearing comfort [39,40]. Considering the microstructure, tensile property, and breathability of TPU ENMs, the sample with a spinning time of three hours was selected as the support layer. 

During the preparation process of this highly sensitive capacitive pressure sensor, one of the critical steps is the synthesis of AgNWs with a suitable morphology and length–diameter ratio. In Figure S3, the SEM and diameter distribution of the synthesized AgNWs in our work are displayed. By adjusting the amount of AgNO_3_ and adding time, it can be clearly seen that the length of the prepared AgNWs was about 500~600 nm and the average diameter was 96.7 ± 3.0 nm. On account of the uniform diameter and relatively small length–diameter ratio of AgNWs, it is probable to introduce AgNWs into the TPU ENM matrix used as the composite dielectric layer. In order to explore the appropriate doping amount and distribution of AgNWs in the AgNW/TPU ENMs, we conducted a series of experiments. 

**Figure 3:**
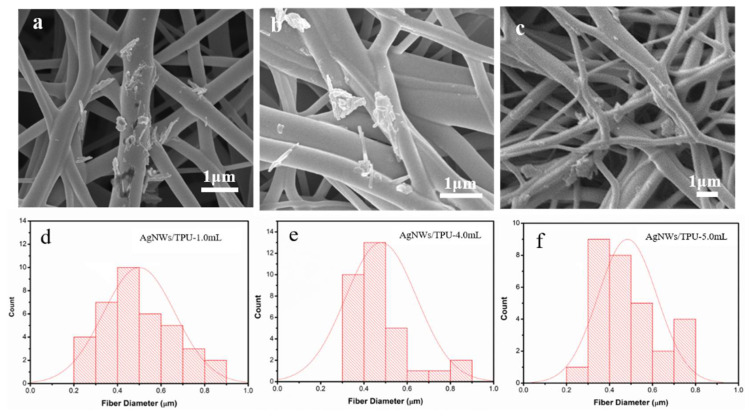
SEM images and fiber diameter distributions of AgNW/TPU composite nanofiber membranes with different AgNW doping amounts: (**a**,**d**) AgNW/TPU-1.0 mL; (**b**,**e**) AgNW/TPU-4.0 mL; (**c**,**f**) AgNW/TPU-5.0 mL.

The SEM images and relevant diameter distributions of AgNW/TPU ENMs with different AgNW doping amounts are shown in Figure 3. As shown in Figure 3a–c, the surface and connection of the TPU nanofibers were covered by AgNWs. Compared with the as-synthesized AgNWs, the length decreased obviously, which may be due to continuous stirring during the preparation of spinning precursors. With the increasing doping content, the AgNWs gradually increased and aggregated in AgNW/TPU-5.0 mL ENMs. Moreover, the fiber diameter of the AgNW/TPU-5.0 mL ENMs exhibited a relatively broad distribution (Figure 3f), while that of the AgNW/TPU-4.0 mL ENMs was in a narrow range (300~500 nm in most cases), which is probably better for the mechanical properties. 

**Figure 4:**
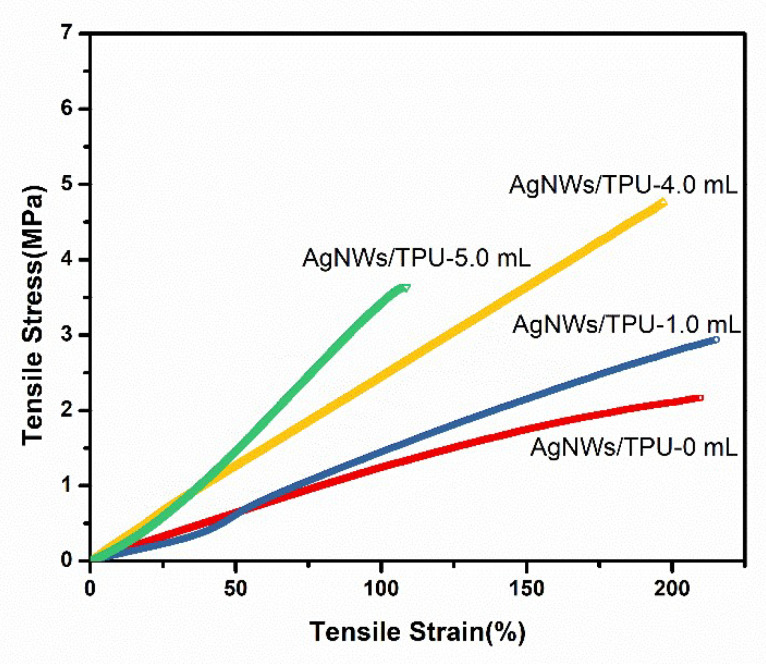
Stress–strain curves of AgNW/TPU ENMs with different AgNWs doping amounts.

As mentioned before, we also needed to test the mechanical properties and thickness (Table S1) of the AgNW/TPU ENMs with different AgNW doping amounts. The stress–strain curves are illustrated in Figure 4. Similar to pure TPU nanofiber membranes, the slope of the stress–strain curves of the composite nanofiber membranes increased continuously with the increase in the addition of AgNWs. Both of the tensile strain and tensile stress went up first by increasing the AgNW doping amount from 0 mL to 5.0 mL, since 1D structured AgNWs act as a reinforcing filler. They were then reduced sharply, because overmuch AgNWs addition depressed the stability of the electrospinning process, resulting in the damage of nanofiber morphology, as illustrated in Figure 3c. Compared to the mechanical properties of the samples with different AgNWs additions, AgNW/TPU-4.0 mL showed excellent performance with a tensile stress of 4.8 MPa and tensile strain of 192%. 

**Figure 5:**
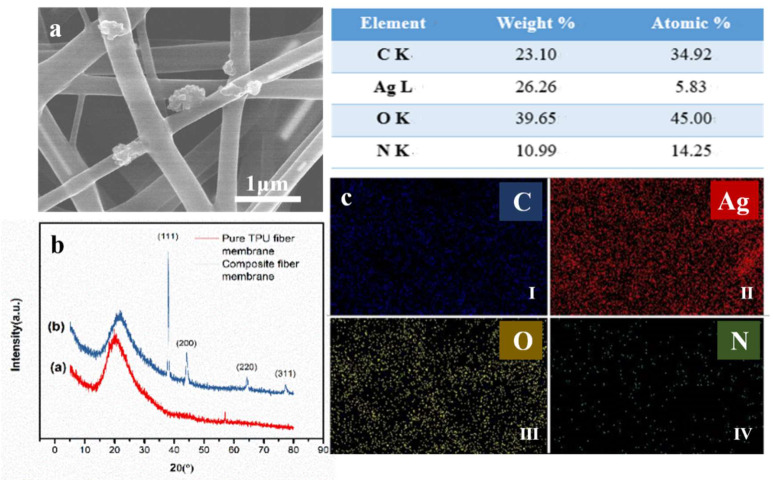
Nanofiber analysis. (**a**) SEM images of the composite nanofiber membrane (AgNW/TPU-4.0 mL), (**b**) The XRD comparison of composite nanofiber membrane (AgNW/TPU-4.0 mL) and pure TPU fiber membrane, (**c**) element mapping analysis of arbitrary interception region of fiber membrane: (I) the location of carbon (blue dots), (II) silver (red dots), (III) oxygen (yellow dots), and (IV) nitrogen (green dots).

Furthermore, aiming to prove the existence of AgNWs in a TPU nanofiber membrane, AgNW/TPU-4.0 mL was characterized by energy-dispersive spectroscopy (EDS) and X-ray diffraction (XRD). In Figure 5, EDS mapping analysis reveals the coexistence of C, O, N, and Ag in the sample, and it can be seen that relative concentration of each individual element was about 23.10, 26.26, 45.00, and 14.25 wt%, respectively. Figure 5 shows the XRD patterns of the pure TPU nanofiber membrane and AgNW/TPU-4.0 mL composite nanofiber membrane. From the curve (a), there was only a wide diffraction peak around 2*θ* = 20° and absence of any other sharp diffraction peak, showing that there was no crystal in the irradiation process of TPU, and that it only had a locally organized structure. In the XRD pattern of the composite nanofiber membrane (curve *b*), it also displayed the wide diffraction peak of TPU around 20° with similar shape. Besides that, the diffraction peak at 2*θ* = 37.95°, 44.19°, 64.39°, and 77.24° corresponded to the characterized diffraction peaks of (111), (200), (220), and (311) for silver (JCPD NO. 99-0094), respectively [41,42,43,44]. 

The formation of an oxide film on the surface of silver by water molecules will reduce its conductivity and subsequently deteriorate the performance of the device. As humidity in the real circumstance is inevitable, the surface layer of the capacitive pressure sensor should be hydrophobic. The water contact angles of AgNW/TPU ENMs with different AgNW doping amounts were measured and are shown in Figure S4. Regardless of the trend, the TPU nanofiber membrane based flexible pressure sensor was still hydrophobic, which could ensure the good running of the sensor under the high humidity. 

### 3.2. Sensing Performances of Flexible Capacitive Pressure Sensor Based on a TPU Nanofiber Membrane

Capacitive pressure sensors are usually designed and assembled in a “sandwich” structure including two flexible outer layers (support layers) with flexible electrodes and a flexible dielectric layer. For this flexible capacitive pressure sensor with a composite dielectric layer, the capacitance was changed by the distance between the two plate electrodes and the permittivity of AgNW/TPU ENMs of the sensor under press and release. 

The flat capacitance (*C*) was calculated from the following formula [33]:

(1) $$C=\frac{\varepsilon s}{d}$$

where *ε* is the permittivity; *s* is the area where the two electrodes overlap; and *d* is the distance between the two plate electrodes. 

The permittivity of the AgNW/TPU ENMs can be calculated from the following formula [28]:

(2) $$\varepsilon =\frac{CD}{\varepsilon_{0}A}$$

where *ε*_0_ is the permittivity of the vacuum (8.854 × 10^−12^ F/m); *D* is the thickness of the dielectric layer; and *A* is the effective sensing area of the pressure sensor. 

The sensitivity (*S*) of the capacitive pressure sensor can be calculated from the following formula [28]:

(3) $$S=\frac{\partial \left(\frac{\Delta C}{C0}\right)}{\partial P}$$

where Δ*C* is the variation in capacitance; *C*_0_ is the capacitance before applying pressure; and *P* is the pressure applied to the sensor. Thus, the sensitivity of the device can be determined by the change in capacitance and the pressure applied. In addition, the response time, detection limit, and long-term stability were also tested using a LCR meter to investigate the sensing performance and mechanism in this work. For comparative study, we first assembled the sensor with TPU-3 h ENMs as the support layers and a pure TPU nanofiber membrane with different spinning times as the dielectric layer. The sensitivity of the pressure sensor prepared by these membranes is shown in Figure S5 and Table S2, which is calculated based on Equation (3). During the experiment, 43 different pressure points were taken from 0 to 50 kPa. It is clear that the sensitivities of the sensor decreased with the increasing spinning time of the dielectric layer. This is because the Young’s modulus of the nanofiber membrane gradually increased with the increase in spinning time, as shown in Figure 2c. As a result, the pressure sensor exhibited less deformation under the same external pressure and the sensitivity declined. 

**Figure 6:**
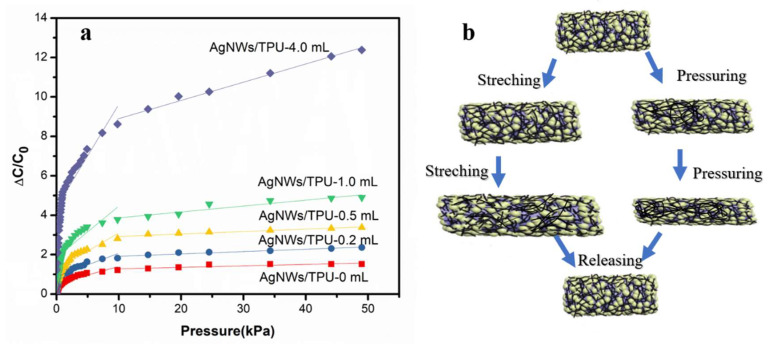
(**a**) Sensitivity test and (**b**) mechanism demonstration of the sensor with AgNW/TPU ENMs as the composite dielectric layer.

In order to further improve the sensitivity of the nanofiber based capacitive pressure sensor, AgNWs were added into the polymer for controlling the permittivity of the dielectric layer. Considering the sensitivity and tensile property of the TPU nanofiber membrane, we chose the nanofiber membrane with a spinning time of three hours to prepare the composite dielectric layer. Figure 6a illustrates the sensitivity of the pressure sensor constructed by the TPU-3 h ENMs as the support layer and AgNW/TPU ENMs as the composite dielectric layer. The sensitivities of the sensors were calculated based on Equation (3) and listed in Table 1. It is obvious that the sensitivities of the sensors increased with an increase in the AgNW doping amount and decreased with increasing external pressure. When the addition of AgNWs was 4.0 mL, *S*_stage1_ was higher than four times the undoped sample (i.e., as high as 7.24 kPa^−1^). The *S*_stage1_ (7.24 kPa^−1^) was higher than the sensor (3.73 kPa^−1^) that Cheng et al. reported [45], which was assembled by hierarchically microstructured Pt/Pyramidal PDMS and Pt/BOPP (biaxially oriented polypropylene) electrodes. In contrast, our sensor achieved an ultrahigh sensitivity in a relatively lower and wider pressure range (7.24 kPa^−1^ at 9.0 × 10^−3^ ~ 0.98 kPa). In spite of this, our sensor still exhibited a high sensitivity of 0.15 kPa^−1^ at a large pressure of 49 kPa. 

To explore the reasons for the results above-mentioned, we calculated the permittivity of the AgNW/TPUENMs that act as the dielectric layer according to Equation (2), as shown in Figure S6. It can be clearly observed that the permittivity of the membrane changed with the addition of AgNWs. As the AgNW doping amount increased from 0 to 1.0 mL, the permittivity of the AgNW/TPU composite nanofiber membrane increased from 0.32 to 0.92, and the sensitivity increased 0.04 kPa^−1^. This indicates that the increased permittivity of the dielectric layer is beneficial to enhance the sensitivity of the capacitive pressure sensor, which was in accordance with the work of Shi et al. [29]. Combined with the calculation values and microstructure, we proposed a theoretical model (Figure 6b), which explained the synergistic effect of the microstructure of the nanofiber membrane and permittivity adjustment of the AgNWs for sensitivity. First, as schematically shown in Figure 6b, when pressure was applied, the interlayer space between the nanofiber layers decreased and caused the first capacitive change (stage 1). As the pressure increased continuously, the fiber-network structure was generally compressed and the interlayer space decreased further, leading to a reduction in the distance between the randomly distributed AgNWs, which in turn led to an increment in the permittivity and further capacitive change (stage 2). Therefore, the sensitivity and detection limit were significantly improved with the nanofiber microstructure and addition of AgNWs. Due to the elastic properties of the polyurethane material itself, further reduction in the distance made the pressure range of the sensor larger (stage 3), but took a lot more force. It is worth mentioning that during the stretching and bending deformation behavior, the change trends of the interlayer space between the nanofiber layers and distance between the AgNWs were similar to the pressing deformation. This indicates that this nanofiber membrane based flexible pressure sensor could also be used in sensing stretching and bending deformation, highlighting the application potential in reality. 

**Figure 7:**
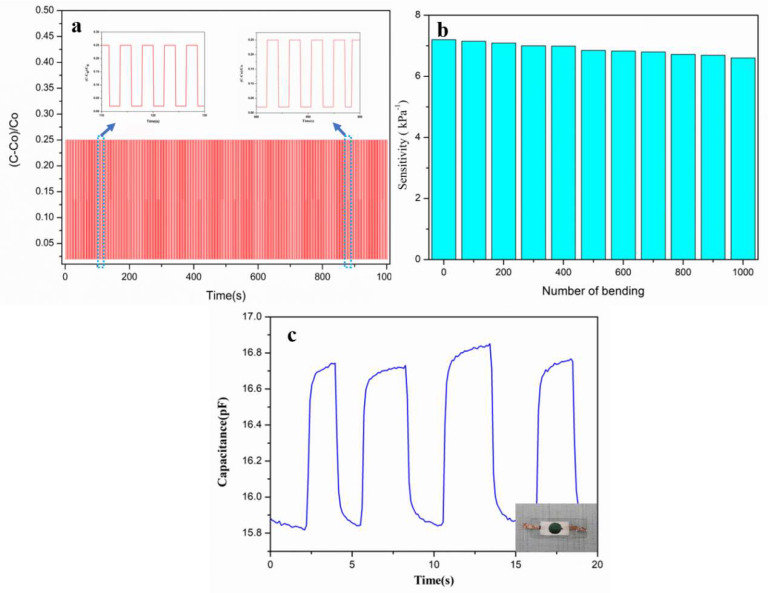
Performance test of the sensor. (**a**,**b**) Mechanical durability and stability test of the prepared sensor, and (**c**) changes in the capacitance of this sensor by loading a Kalanchoe blossfeldiana leaf.

In light of their practical application, the durability, stability, and highly-sensitivity were tested. The pressing-releasing tests (1000 s) at 1.0 kHz under a constant external pressure of 125 Pa are depicted in Figure 7a. It is worth noting that the sensor had excellent mechanical durability and stability against repeated tests. The enlarged image shows the almost identical responses at different times. As illustrated in Figure 7a, it can be seen that the response time of the sensor was about 55 ms. At the same time, we bent the sensor at different times and tested the sensitivity after bending, as shown in Figure 7b, where the sensitivity of the sensor without bending was about 7.24 kPa^−1^ and after bending the sensor 1000 times, the sensitivity was about 6.95 kPa^−1^, indicating that the sensor had good stability and mechanical properties. Next, we loaded a plant (*Kalanchoe blossfeldiana*) leaf of 0.3772 g on the sensor to test the detection limit. It can be observed from Figure 7c that the relative capacitance of the sensor changed from 0.07 pF to 0.13 pF after the leaf was placed. The pressure (about 9.24 Pa) provided by the leaf was very small, but the capacitance changed obviously, which means the very low detection limit of our sensor. 

## 4. Conclusions

In summary, a highly-sensitive and durable capacitive pressure sensor that is completely assembled by elastomer electrospinning nanofiber membranes with excellent breathability and flexibility, was successfully fabricated via electrospinning technology and the screen printing method. By changing the amount of AgNWs, the sensor exhibited a low detection limit (9.24 Pa), high sensitivity (7.24 kPa^−1^ within the range of 9.0 × 10^−3^ Pa ~ 0.98 kPa) and remarkable breathability as well as fast responsive time (55 ms). Moreover, after pressing/releasing over 1000 s and bending over 1000 times, the sensitivity of our sensor had hardly been affected, demonstrating its stability and durability. Furthermore, these stretchable, highly sensitive AgNW/TPU ENMs fabricated by an easy and scalable method make them promising for use in electronic skins and wearable devices. At the same time, the small pressure provided by the leaf (about 9.24 Pa) caused a significant change in the capacitance of sensor, proving that our sensor has a very low detection limit. 

## Figures and Tables

**Table 1:** Sensitivity of sensor with AgNW/TPU ENMs as the dielectric layer.

	Pressure Stage	(Stage1) 9.0 × 10^−3^ ~ 0.98 kPa	(Stage2) 0.98 ~ 9.8 kPa	(Stage3) 9.8 ~ 49 kPa
Sample				
AgNWs/TPU-0.2 mL		2.02 kPa^−1^	0.19 kPa^−1^	0.03 kPa^−1^
AgNWs/TPU-0.5 mL		2.06 kPa^−1^	0.21 kPa^−1^	0.03 kPa^−1^
AgNWs/TPU-1.0 mL		2.74 kPa^−1^	0.26 kPa^−1^	0.04 kPa^−1^
AgNWs/TPU-4.0 mL		7.24 kPa^−1^	0.52 kPa^−1^	0.15 kPa^−1^

## Supplementary Materials

The following are available online at https://www.mdpi.com/1424-8220/20/9/2459/s1, The supplementary parts of the article are put in the supplementary materials file.
