# Supplementary material for: Highly Sensitive, Breathable, and Flexible Pressure Sensor Based on Electrospun Membrane with Assistance of AgNW/TPU as Composite Dielectric Layer
Source: Sensors (Basel). 2020 Apr 26;20(9):2459. doi: 10.3390/s20092459 (PMC7273205; doi:10.3390/s20092459)
Supplement: Supplementary file 1 [file sensors-20-02459-s001.pdf]

Highly Sensitive, Breathable, and Flexible Pressure Sensor Based on Electrospun Membrane with Assistance of AgNW/TPU as Composite Dielectric Layer

Jie Wang [1], Yaoyuan Lou [1], Bin Wang [1,2,*], Qing Sun [3], Mingwei Zhou [1] and Xiuyan Li [1]

[1] School of Materials Science & Engineering, Beijing Institute of Fashion Technology, Beijing 100029, China; 15313719909@163.com (J.W.); clylxyan@bift.edu.cn (X.L.)

[2] Beijing Key Laboratory of Clothing Materials R & D and Assessment, Beijing Engineering Research Center of Textile Nanofiber, Beijing Institute of Fashion Technology, Beijing 100029, China

[3] College of Materials Science and Engineering, Zhejiang University of Technology, Hangzhou 310014, Zhejiang, China

* Correspondence: 20150010@bift.edu.cn (B.W.); Tel.: +86 18612268992

Supplementary Materials

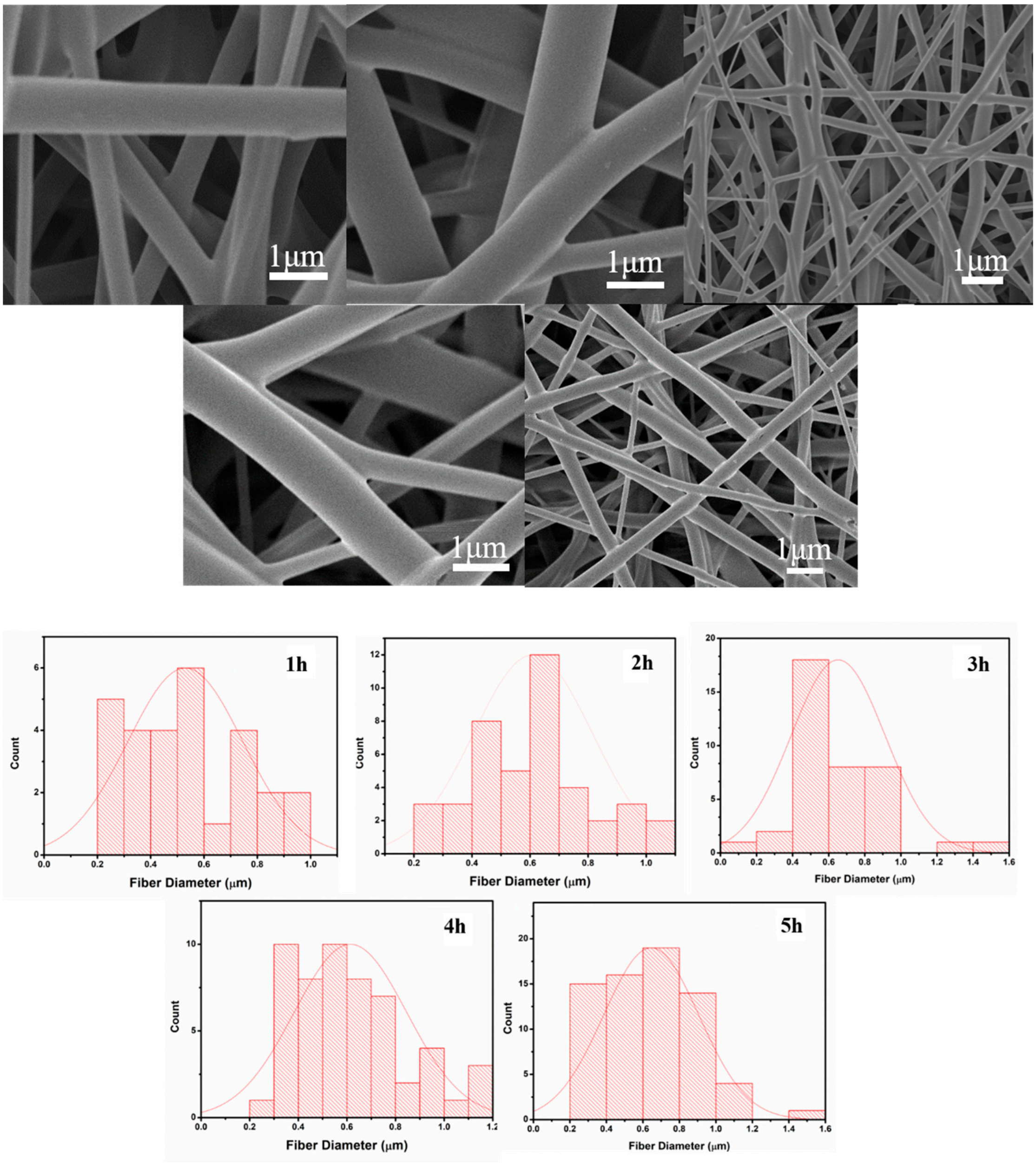

Figure S1. Scanning electron microscopy (SEM) images and diameter distributions of the Thermoplastic polyurethanes (TPU nanofiber membranes with spinning time of 1~5 h.

The SEM images of TPU nanofiber membranes with different spinning times are shown in Figure S1. The SEM shows that the morphology of the prepared TPU nanofiber was smooth and had a good fibrous structure. ***SmileView*** software was used to calculate the diameter and the corresponding distribution of the nanofibers. The average diameters of the TPU nanofibers with spinning time from 1 h to 5 h were about 530 nm, 610nm, 652 nm, 614 nm, and 646 nm, respectively. The results showed that the diameter of the TPU nanofibers increased with the extension of the spinning time. When the spinning time exceeded three hours, the average diameter of the nanofibers changed little, as shown in Figure S1.

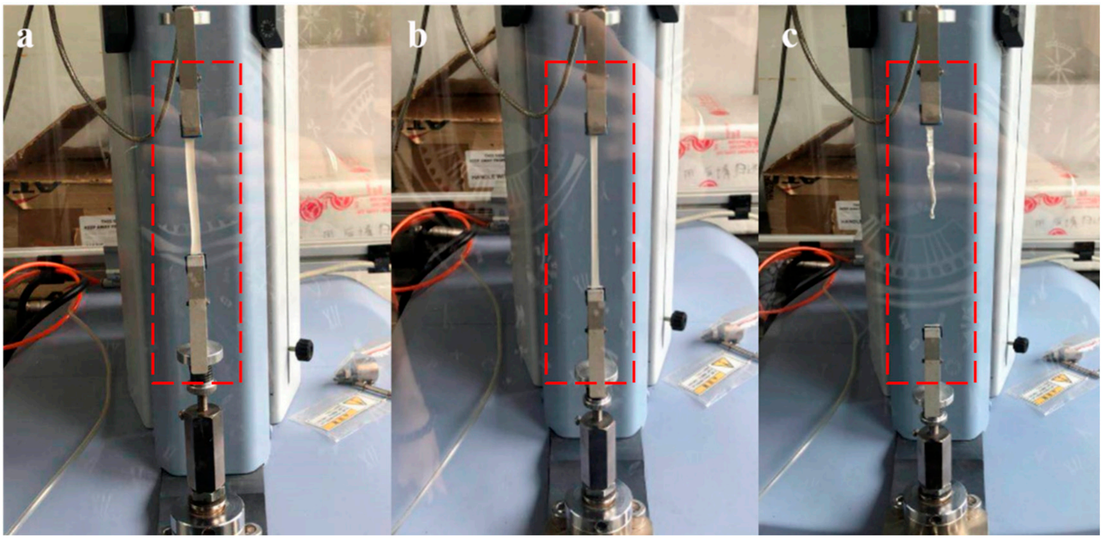

Figure S2. Test process of the tensile properties.

The test process of the TPU electrospinning nanofiber membranes(ENMs) and AgNW/TPU ENMs' tensile properties is shown in Figure S2. First, the TPU ENMs and AgNW/TPU ENMs were cut into splines of 2 cm × 5 cm and fixed on the instrument. We set the test speed at 50 mm/min under a tension of 8 N. The stretching process extended from the original length of the sample (a) until the tensile fracture occurred (c).

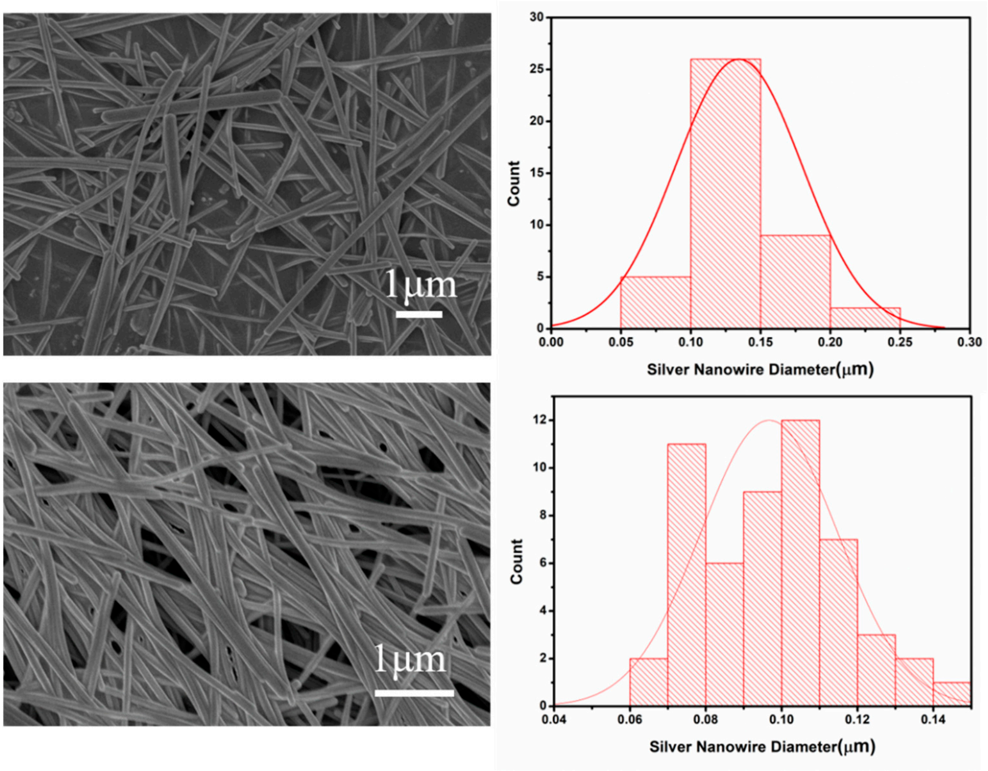

Figure S3. SEM images and diameter distribution of AgNWs.

During the preparation process of a capacitive on-skin pressure sensor, one of the critical steps is the synthesis of AgNWs with a suitable morphology and length–diameter ratio. Figure S3 displays the SEM and diameter distribution of the synthesized AgNWs in our work. By adjusting the amount of $AgNO_3$ and added time, it can clearly be seen that the length of prepared AgNWs was about 500 ~ 600 nm and the average diameter of the AgNWs was about 96.7 ± 3.0 nm. On account of the uniform

diameter and relatively small length–diameter ratio of AgNWs, it is probable to introduce AgNWs into the TPU ENM matrix.

The sample thickness with different spinning times and different AgNWs amounts are shown in Table S1. It can clearly be seen that the thickness of the nanofiber membrane increased by the increasing spinning time at the fixed AgNWs amount. Under the same spinning time, the thickness of the nanofiber membrane gradually increased with the increase in the amounts of AgNWs because the AgNWs attached onto the surface of the TPU fiber and thus increased the thickness of the nanofiber membrane.

Table S1. The sample thickness with different spinning times and different amounts of AgNWs.

**Thickness Time (μm)** / **AgNWs Amount**	**1 h**	**2 h**	**3 h**	**4 h**	**5 h**
0 mL	13.5	23.7	35.6	39.7	51.4
0.5 mL	21.7	29.7	43.6	49.5	60.9
1.0 mL	28.6	36.4	50.4	60.6	68.3
4.0 mL	40.3	43.2	59.4	71.2	83.1

In order to prove the hydrophobicity of the nanofiber membrane, the test results of water contact angle are shown in Figure S4. The sample of pure TPU ENMs, AgNW/TPU-0.5 mL, AgNW/TPU-1.0 mL, and AgNW/TPU-4.0 mL ENMs were 112.4°, 103.2°, 107.6°, and 123.7°, respectively. Regardless of the trend, the TPU nanofiber membrane based on-skin pressure sensor was still hydrophobic, which could ensure the good running of the sensor under high humidity circumstances.

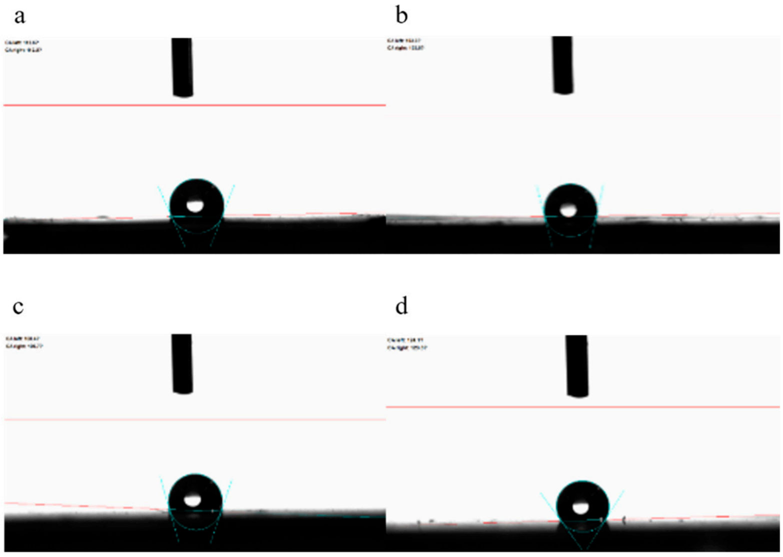

Figure S4. The water contact angles of the AgNW/TPU composite nanofiber membranes with different AgNWs doping amounts: (**a**) AgNW/TPU-0 mL, (**b**) AgNW/TPU-0.5 mL, (**c**) AgNW/TPU-1.0 mL, (**d**) AgNW/TPU-4.0 mL.

The sensitivity of the pressure sensor prepared by these membranes is shown in Figure S5a and Table S2. The curves were divided into three stages for linear fitting, in which the first stage was 9.0 × 10^{-3} kPa ~ 0.98 kPa ($S_{\text{stage 1}}$), the second stage was 0.98 kPa ~ 9.8 kPa ($S_{\text{stage 2}}$), and the third stage was 9.8 kPa ~ 49 kPa ($S_{\text{stage 3}}$). It is clear that the sensitivities of the sensor decreased with the increasing spinning time of the dielectric layer, and $G_{\text{stage 1}}$ was found to be as high as 4.97 kPa^{-1} of TPU-1 h as dielectric layer.

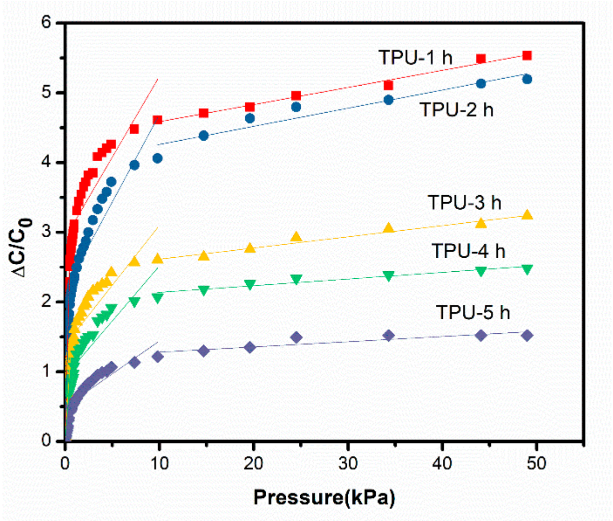

Figure S5. The sensitivity of the sensor with TPU ENMs as the dielectric layer.

Table S2. The sensitivity of the sensor with TPU ENMs as the dielectric layer.

Pressure Stage / Sample	(Stage 1) 9.0 × 10^{-3} ~ 0.98 kPa	(Stage 2) 0.98 ~ 9.8 kPa	(Stage 3) 9.8 ~ 49 kPa
TPU-1 h	4.97 kPa^{-1}	0.38 kPa^{-1}	0.03 kPa^{-1}
TPU-2 h	3.58 kPa^{-1}	0.25 kPa^{-1}	0.03 kPa^{-1}
TPU-3 h	1.74 kPa^{-1}	0.14 kPa^{-1}	0.02 kPa^{-1}
TPU-4 h	1.11 kPa^{-1}	0.14 kPa^{-1}	0.01 kPa^{-1}
TPU-5 h	0.69 kPa^{-1}	0.08 kPa^{-1}	0.01 kPa^{-1}

The permittivity of the AgNW/TPU ENMs, which acts as a dielectric layer according to Equation (2), as shown in Figure S6. It can be clearly seen that the permittivity of the membrane changed with the addition of the AgNWs. With the AgNWs doping amount increased from 0 to 1.0 mL, the permittivity of the AgNW/TPU composite nanofiber membrane increased from 0.32 to 0.92, and the sensitivity increased 0.04 kPa^{-1}.

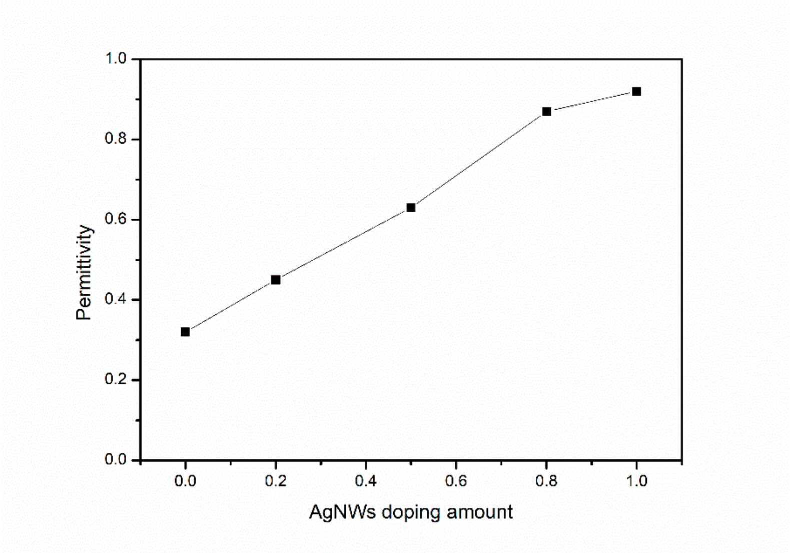

Figure S6. The influence of the AgNWs doping amount on permittivity.
